# Metronidazole Degradation by UV and UV/H_2_O_2_ Advanced Oxidation Processes: Kinetics, Mechanisms, and Effects of Natural Water Matrices

**DOI:** 10.3390/ijerph191912354

**Published:** 2022-09-28

**Authors:** Rongkui Su, Xiangrong Dai, Hanqing Wang, Zhixiang Wang, Zishi Li, Yonghua Chen, Yiting Luo, Danxia Ouyang

**Affiliations:** 1 PowerChina Zhongnan Engineering Corporation Limited, Changsha 410004, China; 2 Hunan First Normal University, Changsha 410114, China; 3 College of Environmental Science and Engineering, Central South University of Forestry & Technology, Changsha 410004, China

**Keywords:** hydroxyl radical, metronidazole, antibiotic, steady-state kinetic model, complex matrix

## Abstract

Advanced oxidation technology represented by hydroxyl radicals has great potential to remove residual antibiotics. In this study, we systematically compared the metronidazole (MTZ) degradation behavior and mechanism in the UV and UV/H_2_O_2_ systems at pH 3.00 condition. The results show that the initial reaction rates were 0.147 and 1.47 µM min^−1^ in the UV and UV/H_2_O_2_ systems, respectively. The main reason for the slow direct photolysis of MTZ is the relatively low molar absorption coefficient (2645.44 M^−1^ cm^−1^) and quantum yield (5.9 × 10^−3^ mol Einstein^−1^). Then, we measured kMTZ,OH • as 2.79 (±0.12) × 10^9^ M^−1^ s^−1^ by competitive kinetics, and calculated kMTZ,OH • and [OH •]SS as 2.43 (±0.11) × 10^9^ M^−1^ s^−1^ and 2.36 × 10^−13^ M by establishing a kinetic model based on the steady-state hypothesis in our UV/H_2_O_2_ system. The contribution of direct photolysis and ^•^OH to the MTZ degradation was 9.9% and 90.1%. ^•^OH plays a major role in the MTZ degradation, and ^•^OH was the main active material in the UV/H_2_O_2_ system. This result was also confirmed by MTZ degradation and radicals’ identification experiments. MTZ degradation increases with H_2_O_2_ dosage, but excessive H_2_O_2_ had the opposite effect. A complex matrix has influence on MTZ degradation. Organic matter could inhibit the degradation of MTZ, and the quenching of the radical was the main reason. ${\mathrm{NO}}_{3}^{-}$ promoted the MTZ degradation, while ${\mathrm{SO}}_{4}^{2-}$ and Cl^−^ had no effect. These results are of fundamental and practical importance in understanding the MTZ degradation, and to help select preferred processes for the optimal removal of antibiotics in natural water bodies, such as rivers, lakes, and groundwater

## 1. Introduction

In recent years, residual antibiotics in urban sewage have become one of the practical problems in the field of sewage treatment technology [1,2]. Antibiotics are a class of organic pollutants closely related to human beings, which will enter the environmental water body through the sewage treatment system [3,4]. At present, there are many reports of residual antibiotics detected in different environmental areas, such as sewage treatment plants, livestock farms, rivers and sediments, soil, and groundwater [5,6,7,8]. The test results of Ben et al. [9] on antibiotics in drinking water and bottled/barreled water showed that at least 5 kinds of antibiotics were detected in each sample of drinking water, and the highest was 41 kinds, with a concentration range of 0.021–1133 ng/L. At least 3 kinds of antibiotics were detected in each sample of bottled/barreled water, up to 31 kinds, and the concentration was between 0.0094 and 3462 ng/L [9]. These residual antibiotics usually have stable chemical properties, a long residual time, low environmental concentration and are not easy to be degraded in the environment, which seriously threatens the safety of food and drinking water, endangers the health of non-target organisms and enhances bacterial resistance [10,11,12]. The drug resistance genes of drug-resistant bacteria can also continue to spread among bacteria, animals and humans, endangering human health [13,14,15]. In the special period when the world is facing the threat of various viruses and bacteria, the use of drugs, especially antibiotics, has increased significantly, which will lead to a sharp increase in the content of residual antibiotics in urban sewage. In view of the continuous increase in antibiotic residues and its adverse impact on the ecological environment and human health, it is urgent to control the residual antibiotics in the environment.

However, many sewage treatment plants adopt the traditional sewage treatment process, which lacks pertinence to this new type of organic pollutants and is difficult to achieve the purpose of effective removal [16,17,18,19,20]. Osińska et al. [16] found that the design operation mode and water treatment process of most sewage treatment plants could not completely remove the antibiotic organic pollutants. Zhao et al. [17] reported that the activated sludge process could not remove trimethoprim well. Jia et al. [21] found that the anaerobic anoxic aerobic treatment unit of the municipal sewage treatment plant had very little effect on the reduction in the quality change percentage of quinolones and fluoroquinolones in the sewage, indicating that biodegradation has little effect on the removal of quinolones and fluoroquinolones in the municipal sewage. Due to the characteristics of antibiotic organic compounds such as water solubility, weak volatility, stable chemical quality, poor biodegradability and high biological toxicity, the traditional flocculation sedimentation adsorption and biodegradation processes cannot effectively and completely remove antibiotics from urban sewage [22,23,24]. Therefore, efficient removal of residual antibiotics in sewage has become a difficult problem to be solved in the process of urban sewage treatment.

Advanced oxidation technologies (AOPs) with hydroxyl radicals (^•^OH) as the main active material is new treatment technologies that improve the biodegradability of wastewater through oxidation or directly degrade organic pollutants through mineralization [25,26,27]. Hydroxyl radical has a high redox potential (*E*_0_ = 1.89~2.72 V vs. NHE), and it is a strong oxidant and has a good degradation effect on organic pollutants. In 1894, Fenton first discovered that the mixed solution of H_2_O_2_ and Fe^2+^ could rapidly oxidize tartaric acid, marking the origin of advanced oxidation technology. In Fenton reagent, H_2_O_2_ is catalytically decomposed by Fe^2+^ ion to generate ^•^OH, which has a strong oxidation ability. Canadian scientist Eisenhaner first applied Fenton reagent to water treatment in 1964, and then a large number of studies expanded its application scope. Traditional AOPs use ^•^OH as the main active material to degrade pollutants. In addition to Fenton reagent and Fenton-like reagent methods, ozone and combined process methods, semiconductor photocatalytic oxidation methods, ferrate oxidation methods, etc., are also common in ^•^OH generation technologies in advanced oxidation [25,28,29,30]. Among them, ^•^OH generated by ultraviolet-activated H_2_O_2_ technology degrades organic pollutants, with the characteristics of simple operation, convenience and efficiency, no special requirements for the environment, less secondary pollution and broad application prospects [31]. However, advanced oxidation technology for the degradation of emerging organic pollutants by ultraviolet-activated hydrogen peroxide still has problems, such as it being difficult to determine the degradation mechanism of pollutants and difficult to predict the impact of the complex matrix.

In this study, we systematically compared the antibiotic degradation behavior and mechanism in the alone UV and UV/H_2_O_2_ systems at pH 3.00 condition. We determined the effective light intensity and the effective light path of the photochemical reaction system by using two chemical photometric methods of potassium ferric oxalate and hydrogen peroxide. Due to its huge usage and environmental relevance, metronidazole (MTZ) was selected as an example of antibiotics in this study [32,33]. Then, we detected the molar absorption coefficient and quantum yield of MTZ by direct UV experiment. MTZ degradation kinetics of the UV and UV/H_2_O_2_ systems was compared. We experimentally measured kMTZ,OH •, [OH •]SS, the contribution of direct photolysis and ^•^OH using the steady-state approximation kinetic model, and kMTZ,OH • was verified by the competitive kinetic method [34,35]. Finally, we studied the effect of the H_2_O_2_ dosage and complex matrix of the MTZ degradation in the H_2_O_2_ system by the steady-state kinetic model and experimentation. The study on the oxidative degradation mechanism and complex matrix effect of the antibiotic is of scientific and practical importance, as those determine the removal efficiency of the antibiotic, where results can vary from partial remediation to complete mineralization.

## 2. Materials and Methods

### 2.1. Materials and Experimental Design

Disodium hydrogen phosphate (99.0%), phosphoric acid (85–90%), sodium dihydrogen phosphate (99.0%), metronidazole (99.0%), 4-chlorobenzoic acid (*p*CBA, 99.0%), t-butanol (99.7%) and fulvic acid (technical) were purchased from Sigma Aldrich. Methanol (chromatographically pure) and acetonitrile (chromatographically pure) were purchased from Merck. Hydrogen peroxide (30% by weight), concentrated sulfuric acid (superior purity), KMnO_4_ (analytical purity), Na_2_C_2_O_4_ (analytical purity), humic acid (analytical purity) was purchased from Sinopharm Chemical Reagent. Other chemical substances involved in the experiment are all superior pure. Deionized (DI) water is prepared by Molresearc 1020A ultrapure water reactor.

Stock solutions of MTZ and *p*CBA were prepared in DI water and stored at 4 °C in the dark. For the kinetic studies, the initial concentrations of MTZ and *p*CBA in the working solutions were 10 μM. Solution pH was adjusted to pH 3.0 with 10 mM phosphate buffer system. The pH value of the reaction system remained constant throughout the experiment. Experimental design and operation process had been introduced in our previous papers [36,37].

### 2.2. Analytical Methods

Average light intensity per volume (*I*_0_) and effective optical path length (*l*) was measured by potassium ferric oxalate and H_2_O_2_ chemical photometer method. USB 2000+, Ocean Optics fiber optic spectrometer was used to measure the emission spectrum and light intensity of low-pressure mercury lamp. Absorption spectra of MTZ and *p*CBA were measured by Shimadzu UV-2550 spectrometer. Solution pH was determined by Mettler Toledo S400-K pH meter. Ultra-performance liquid chromatography (UPLC, Waters ACQUITY H-Class, Waters, Milford, MA, USA), which has a BEH C18 column (1.7 μm, 2.1 mm × 50 mm, Waters, Milford, MA, USA), was used to analyze MTZ and *p*CBA content. Detailed analysis method is shown in Table 1.

### 2.3. Statistics Analysis

Data were statistically analyzed by using Microsoft Office Excel 2016. Charts are drawn by Origan Pro 2018. All data were expressed as mean ± standard deviation of three replicate experiments (n = 3).

## 3. Results and Discussion

### 3.1. Effective Light Intensity and Optical Path of Photochemical Reaction System

Chemical photometry is a chemical method widely used in photochemistry research to measure the irradiation intensity of photochemical systems [38,39,40,41]. Compared with other measurement methods, the chemical photometer measurement method has the characteristics of simple operation, sensitive response, reliable data, and so on. It can accurately measure the radiation intensity and has good reproducibility [39,40]. Therefore, as a simple and accurate method to determine the irradiation intensity, chemical photometry has been widely used in the field of photochemistry in recent years.

The chemical photometry method is mainly based on the principle of photochemical reaction, and selects substances whose quantum yield has been accurately measured and whose value is relatively stable to measure the irradiation intensity of the photochemical system [38,39]. Substances selected by the chemical photometry method may not be limited by the form. At present, liquid-phase chemical photometry is more used in the determination of irradiation intensity of the photochemical system, while gas-phase and solid-phase chemical photometry are less used [38,40]. Gas phase chemical photometry is mainly used to measure the irradiation intensity in a vacuum system [40,41].

In this study, the effective irradiation intensity of the low-pressure mercury lamp in the photochemical system was measured by the potassium ferric oxalate chemical photometer method [38,39] and the hydrogen peroxide chemical photometer method [40,41,42]. Both of the two chemical photometry methods belong to the liquid phase chemical photometry method, which is mainly applicable to the light source with the wavelength range of 200~380 nm. The main irradiation intensity of the low-pressure mercury lamp used in this study is concentrated at 254 nm, which is included in the measurement wavelength range of potassium ferric oxalate and hydrogen peroxide chemical photometer method.

#### 3.1.1. Potassium Ferric Oxalate Chemical Photometer Method

In this method, the absorbance of the red complex formed by the reaction of analytical reagent 1, 10 o-phenanthroline with Fe^2+^ was measured by UV-Vis spectrophotometer at 510 nm, and the concentration of Fe^2+^ was calculated according to the standard curve. Fe^2+^ is produced by Fe^3+^ under acidic conditions through light radiation. The stoichiometric relationship of the reaction is as follows [38]:

(1) $$2\;{\left[\mathrm{Fe}{\left(C_{2}O_{4}\right)}_{3}\right]}^{3-}+hv\to \;2\;{\left[\mathrm{Fe}{\left(C_{2}O_{4}\right)}_{2}\right]}^{2-}{+[C}_{2}O_{4}{]}^{2-}{+2\mathrm{CO}}_{2}$$

Effective light intensity can be calculated according to the following two methods:

(1) Calculate the fitting equation of $\left[{\mathrm{Fe}}^{2+}\right]$ for UV irradiation time *t*, and obtain $k_{0}$ value.
  (2) $$\frac{d\left[{\mathrm{Fe}}^{2+}\right]}{\mathrm{dt}}{=k}_{0}$$

Effective radiation intensity can be calculated by the following formula:

(3) $$I_{0}{=k}_{0}/\Phi$$

where $I_{0}$ is the effective light intensity (Einstein L^−1^s^−1^); *Φ* is the quantum yield of potassium iron oxalate, and the quantum yield at 254 nm is 1.25 mol Einstein^−1^ [43].

(2) Effective light intensity ($I_{0}$) can also be calculated by the following formula:

(4) $$I_{0}=\Delta n/(10^{-3}\Phi V_{1}t)$$

where $I_{0}$ is the effective light intensity (Einstein L^−1^s^−1^); Δn is the amount of Fe^2+^ generated at time *t* of photochemical reaction (mole); *Φ* is the quantum yield of potassium iron oxalate, and the quantum yield at 254 nm is 1.25 mol Einstein^−1^ [43]; V_1_ is the volume of reaction solution exposed to light (mL); *t* is the reaction time (s).

Δn can be calculated by the following formula:

Δn = 10^−3^V_1_V_3_*C*_t_/V_2_ (5)

where V_1_ is the volume of reaction solution illuminate; V_2_ is the volume of sampling and analysis (mL); V_3_ is the total volume of absorbance measured after dilution (mL); *C*_t_ is the concentration of Fe^2+^ measured after dilution (M).

*C*_t_ can be calculated according to the following formula by measuring the absorbance at 510 nm:

*C*_t_ = *A*/(*ε*·*l*) (6)

where *A* is the absorbance at 510 nm; *ε* is the molar absorption coefficient (M^−1^ cm^−1^), which is equal to the slope of the previous standard curve; *l* is 1 cm, the optical path of the cuvette.

**Figure 1:**
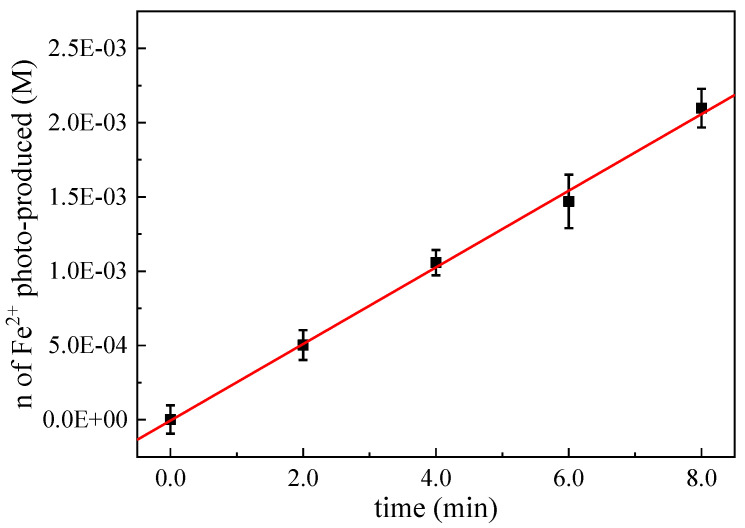
Potassium oxalate method for the determination of effective light intensity.

According to the experimental results, the fitting curve of the amount of photogenerated Fe^2+^ to the illumination time is drawn, as shown in Figure 1.

According to the above calculation method of effective irradiation intensity, the average value of the measured effective irradiation intensity is 7.50 (±0.23) × 10^−6^ Einstein L^−1^ s^−1^. When the interval between two batches of experiments is too long, the effective light intensity needs to be re-measured to eliminate interference.

#### 3.1.2. Hydrogen Peroxide Chemical Photometer Method

Based on quantum yield and Lambert–Beer law, the overall photolysis rate of hydrogen peroxide can be described as:

(7) $$-\frac{\mathrm{dC}}{\mathrm{dt}}{=\Phi }_{\lambda }I_{0}(1-e^{-{2.303\varepsilon }_{\lambda }lC})$$

where $\Phi_{\lambda }$ is the quantum yield of hydrogen peroxide at 254 nm (0.5 mol Einstein^−1^); $I_{0}$ is the effective light intensity (Einstein L^−1^ s^−1^); $\varepsilon_{\lambda }$ is the molar absorption coefficient (M^−1^ cm^−1^), and the molar absorption coefficient of hydrogen peroxide at 254 nm is 19.6 M^−1^ cm^−1^; *l* is the effective optical path (cm); C is the initial concentration of hydrogen peroxide (M).

When 2.303*ε*_λ_*lC* > 2, this formula can be simplified as

(8) $$-\frac{\mathrm{dC}}{\mathrm{dt}}{=\Phi }_{\lambda }I_{0}$$

When 2.303*ε*_λ_*lC* < 0.02, this formula can be simplified as

(9) $$-\frac{\mathrm{dC}}{\mathrm{dt}}=2.303\Phi_{\lambda }I_{0}\varepsilon_{\lambda }lC$$

This simplified formula can be used to calculate the effective light intensity and effective optical path of the photochemical reaction system, respectively [41].

**Figure 2:**
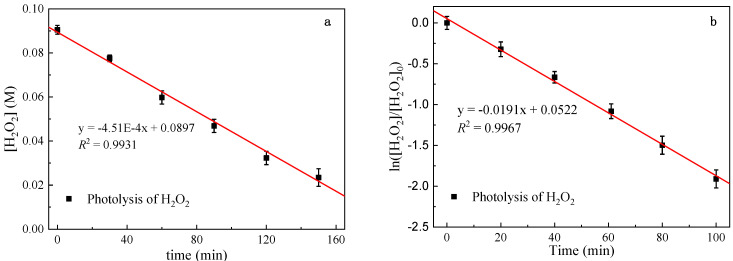
High (**a**) and low (**b**) concentration photolysis of H_2_O_2_.

As shown in Figure 2, the photodegradation experimental results of high-concentration H_2_O_2_ in the photochemical reaction system are fitted to the zero-order reaction kinetics, and the *R*^2^ was 0.9931. According to the above light intensity calculation formula, the effective light intensity calculation result is 7.50 (±0.25) × 10^−6^ Einstein L^−1^ s^−1^. The result is consistent with the value determined by potassium ferric oxalate photometry, proving that the two methods have good repeatability and effectiveness.

Photodegradation experimental results of low concentration H_2_O_2_ in the photochemical reaction system shows that the fitting degree of the first-order reaction kinetics is the highest. Therefore, the photolysis of low-concentration H_2_O_2_ is more in line with the first-order reaction kinetic equation. The effective optical path can be calculated according to the formula *l* = *k*_obs_/2.3*ε Φ*_p_*I*_0_. As shown in the H_2_O_2_ photolysis experimental results (Figure 2b), *R*^2^ fitting the first-order reaction kinetics reaches 0.9967. The effective optical path was measured as 0.935 (±0.04) cm.

The results show that the measured value by the hydrogen peroxide chemical photometer method is consistent with that of the potassium ferric oxalate chemical photometer method, which has good repeatability and effectiveness. It should be noted that when the interval between two batches of experiments is too long, the effective light intensity and the effective optical path of the photochemical reaction system need to be recalibrated to eliminate the interference as much as possible.

### 3.2. Direct Photolysis of Metronidazole

**Figure 3:**
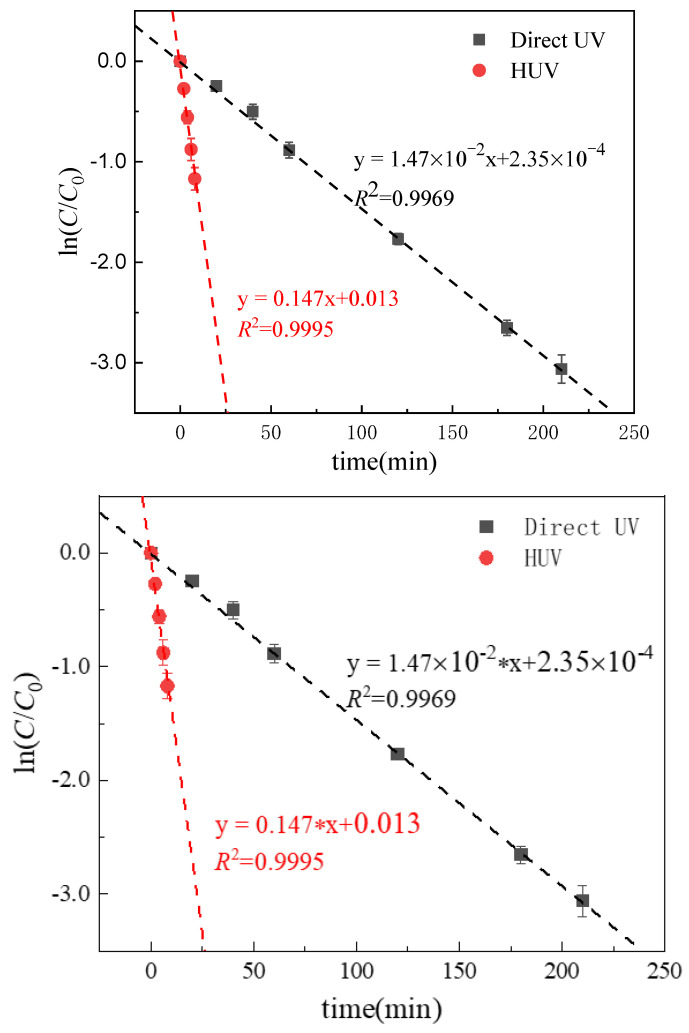
Time-dependent degradation kinetics of MTZ in the UV, UV/H_2_O_2_ system. The degradation was fitted to a first-order kinetic model (lines). ([MTZ] = 10 μM, [H_2_O_2_] = 100 μM, *I*_0_ = 7.50 × 10^−6^ Einstein L^−1^·s^−1^, *l* = 0.935 cm).

The kinetic equation fitting of the direct degradation process of the MTZ-containing nitroimidazole ring structure in the UV system was carried out using the linear regression method, and the results show that the pseudo first-order reaction kinetics were followed (Figure 3). Under the UV radiation of light intensity 7.50 × 10^−6^ Einstein L^−1^s^−1^, the initial direct photolysis rate of MTZ (initial concentration 10 µM) is 0.147 µM min^−1^ (pH = 3.00).

Molar absorption coefficient and quantum yield have important effects on the direct photolysis of compounds [44,45]. Molar absorption coefficient (*ε*) indicates that the compound absorbs a specific wavelength (λ) light. *ε* can be calculated by measuring the absorbance (*A*) of MTZ (10 µM) solution with pH = 3.00 in 1 cm optical path (*l*) quartz cuvette:

(10) A=ε [MTZ]l

**Figure 4:**
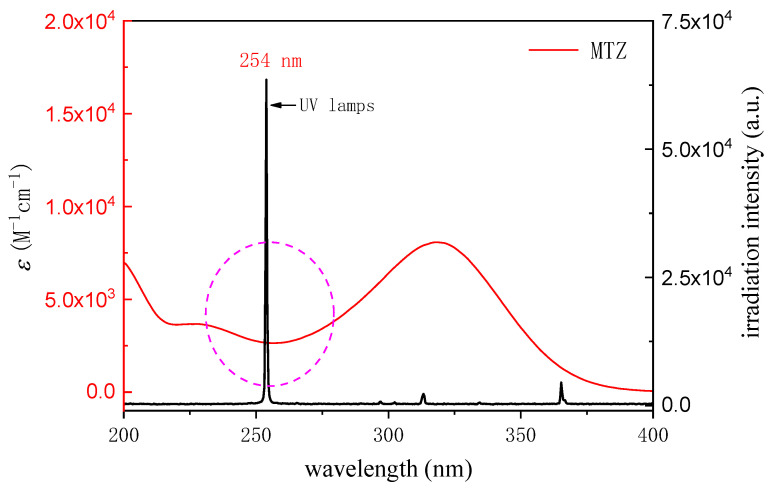
Decadic molar absorption coefficient (*ε*) of MTZ with reference to the UV lamp emission spectra from 200 to 400 nm.

Figure 4 shows the overlapping diagram of the molar absorption coefficient of MTZ and the emission spectrum of the low-pressure mercury lamp. It can be seen that the emission wavelength of the low-pressure mercury lamp (GPH212T5L/4, 10 W, Heraeus) is mainly distributed around 254 nm. The main UV absorption range of MTZ is 300~350 nm, and the UV absorption of 254 nm mainly emitted by the low-pressure mercury lamp used in this experiment is weak. The molar absorption coefficient of MTZ at 254 nm is 2645.44 M^−1^ cm^−1^ (pH 3.00). Previously, the molar absorptivity of MTZ has not been reported yet. Compared with other PPCPs, the molar absorptivity is at a lower level [46]. For example, Kwon et al. [47] reported that the molar absorption coefficient of IBU at 254 nm was 256 M^−1^ cm^−1^ under neutral conditions, Vogna [48] reported that the molar absorption coefficient of CBZ at 254 nm was 6025 M^−1^ cm^−1^, and Yang [46] reported that the molar absorption coefficient of sulfamethoxazole at 254 nm under neutral conditions was 16,200 M^−1^ cm^−1^.

Quantum yield represents the utilization rate of light quantum in photochemical reaction and describes the ratio of the total number of effective photons to the total number of photons absorbed by the compound. The quantum yield of MTZ can be calculated as follows [44]:

(11) $$\varphi_{\mathrm{MTZ}}=\frac{r_{\mathrm{UV}}}{I_{0}(1-10^{-\varepsilon_{\mathrm{MTZ}}\;l\;\left[\mathrm{MTZ}\right]})}$$

where $\varphi_{\mathrm{MTZ}}$ (mol Einstein^−1^) is the quantum yield of MTZ at 254 nm; *r*_uv_ (M s^−1^) is the direct photolysis rate (MTZ initial concentration 10 μM); $\varepsilon_{\mathrm{MTZ}}$ is the molar absorption coefficient at 254 nm (2645.44 M^−1^ cm^−1^, pH 3.00). The $\varphi_{\mathrm{MTZ}}$ (5.9 × 10^−3^ mol Einstein^−1^) is consistent with the reported value 3.3 × 10^−3^ mol Einstein^−1^ [49]. $\varphi_{\mathrm{MTZ}}$ is higher than trimethoprim (1.52 × 10^−3^ mol Einstein^−1^), and close to naproxen (9.3 × 10^−3^ mol Einstein^−1^), but lower than phenytoin sodium (0.279 mol Einstein^−1^) and clofibric acid (0.539 mol Einstein^−1^) [44,50]. Although the molar absorption coefficient of MTZ was 2645.44 M^−1^ cm^−1^, low $\varphi_{\mathrm{MTZ}}$ (5.9 × 10^−3^ mol Einstein^−1^) leads to the low direct photolysis of MTZ.

### 3.3. MTZ Degradation Kinetic in UV/H_2_O_2_ System

The degradation process of MTZ in the UV/H_2_O_2_ system was fitted to the kinetic equation using the linear regression method. The results show that the degradation of MTZ followed the pseudo first-order reaction kinetics (Figure 3). MTZ concentration in the system did not change within 60 min after adding 100 µM H_2_O_2_ to the dark reaction control experiment, indicating that, alone, H_2_O_2_ had no degradation effect on MTZ. Compared with UV irradiation alone, the MTZ degradation rate was significantly increased after adding 100 μM H_2_O_2_ to the UV system, and the degradation rate was 1.47 µM min^−1^ at pH 3.00, which is 10 times higher than that of UV alone. Therefore, free radicals might play a major role in MTZ degradation.

**Figure 5:**
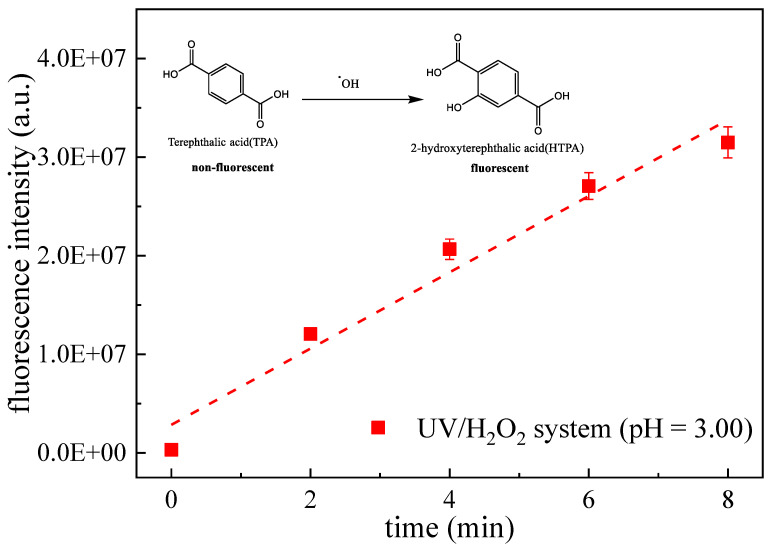
Fluorescence signal intensity of HTPA over time. ([TPA] = 100 μM, [H_2_O_2_] = 100 μM, and *I*_0_ = 7.50 × 10^−6^ Einstein L^−1^ s^−1^).

At present, TPA is often used to measure ^•^OH in reaction systems by fluorimetry [51,52]. TPA itself does not fluoresce, but it can react with ^•^OH to produce 2-hydroxyterephthalic acid (HTPA), which has fluorescence characteristics. As shown in the interior drawing of Figure 5, HTPA is the only product due to the symmetry of the TPA structure.

As shown in Figure 5, ^•^OH exists in the UV/H_2_O_2_ system. Compared with UV alone, the main reason for the MTZ degradation rate increase in the UV/H_2_O_2_ system is that the dominant mechanism of MTZ degradation in the photoreaction system is turn the direct photolysis induced by UV to the ^•^OH-mediated oxidation produced by UV-activated H_2_O_2_.

### 3.4. Competitive Kinetic

Second-order reaction rate constant kMTZ,OH • for ^•^OH and MTZ reactions can be calculated by the competitive kinetic method with 4-chlorobenzoic acid (*p*CBA) as the reference substance, and the calculation formula is as follows [53,54,55]:

(12) kMTZ,OH •kpCBA,OH •=(ln [MTZ]t [MTZ]0)tot−(ln [MTZ]t [MTZ]0)UV(ln [pCBA]t [pCBA]0)tot−(ln [pCBA]t [pCBA]0)UV=ktot, MTZ−kUV, MTZktot, pCBA−kUV, pCBA

From the above formula, $k_{\mathrm{tot},\mathrm{MTZ}}-k_{\mathrm{UV},\mathrm{MTZ}}$ is the MTZ degradation rate caused by ^•^OH, $k_{\mathrm{tot},p\mathrm{CBA}}-k_{\mathrm{UV},p\mathrm{CBA}}$ is the *p*CBA degradation rate caused by ^•^OH. At pH 3.00, the average reaction rate constant ratio of MTZ and *p*CBA to ^•^OH is 0.62 ([MTZ] = [*p*CBA] = 10 μM, [H_2_O_2_] = 100 μM, and *I*_0_ = 7.50 × 10^−6^ Einstein L^−1^ s^−1^, *l* = 0.935 cm), kMTZ,OH • value is calculated as 2.79 (±0.32) × 10^9^ M^−1^ s^−1^. The value of kMTZ,OH • in this study is consistent with the measured value 3.54 (±0.42) × 10^9^ M^−1^ s^−1^, which is reported by Lian [56]. kMTZ,OH • is smaller than that of other compounds with ^•^OH. For example, the *k* value of trimethoprim with ^•^OH is 6.69 × 10^9^ M^−1^ s^−1^ [57], the *k* value of ibuprofen with ^•^OH is 5.57 × 10^9^ M^−1^ s^−1^ [47] and the *k* value of sulfamethoxazole with ^•^OH is 1.2 × 10^10^ M^−1^ s^−1^ [58].

### 3.5. Pseudo First-Order Reaction Kinetic Model Based on Steady State Assumption

MTZ degradation kinetic in the UV/H_2_O_2_ system can be explained by the pseudo first-order reaction kinetic model of free radical steady state hypothesis. This method is based on the assumption that the free radicals (i.e., ^•^OH) generated by UV-excited H_2_O_2_ play a major role in the degradation of the target compound, and the free radical concentration in the system is relatively stable [36]. The main reactions and reaction rate constants in the UV/H_2_O_2_ system are summarized in Table 2. The kinetic model of MTZ degradation in the UV/H_2_O_2_ system is established as follows.

Under steady-state conditions, the reaction rate (*r*_tot_, M s^−1^) of MTZ in the UV/H_2_O_2_ system can be expressed as the following formula [59]:

(13) rtot=rUV+rOH •

where *r*_UV_ is the initial reaction rate of direct photolysis of MTZ in the UV/H_2_O_2_ system; rOH • is the reaction rate of MTZ with ^•^OH. *r*_UV_ and rOH • can be expressed by the following formula:

(14) $$r_{\mathrm{UV}}{=\varphi }_{\mathrm{MTZ}}I_{0}\frac{\;l\;\varepsilon_{\mathrm{MTZ}}\;[\mathrm{MTZ}]}{A}\left({1-10}^{-A}\right)$$

(15) rOH •=kMTZ,OH •[OH •]SS [MTZ]

(16) $$A=l\;(\varepsilon_{\mathrm{MTZ}}\;[\mathrm{MTZ}]+\varepsilon_{H_{2}O_{2}}\;[H_{2}O_{2}])$$

where *I*_0_ is the effective light intensity; l is the effective optical path; *A* is the absorbance value of the reaction solution; $\varepsilon_{H_{2}O_{2}}$ is the molar absorptivity of H_2_O_2_ at 254 nm (19.6 M^−1^ cm^−1^) [59]. $\varepsilon_{\mathrm{MTZ}}$ is the molar absorption coefficient of MTZ at 254 nm; $\varphi_{\mathrm{MTZ}}$ is the quantum yield of MTZ at 254 nm; [OH •]SS is the steady-state concentration of ^•^OH in the system; kMTZ,OH • are the second-order reaction rate constants of MTZ and ^•^OH. In this system, *I*_0_ is 7.50 × 10^−6^ Einstein L^−1^ s^−1^, l is 0.935 cm, the molar absorptivity of the target compound MTZ in this study is 2645.44 M^−1^ cm^−1^, and the quantum yield is 5.9 × 10^−3^ mol Einstein^−1^ (pH 3.00). [MTZ]_0_ and [H_2_O_2_]_0_ are actually measured as 10 and 90 μM, respectively. The reaction condition was stable at pH 3.00.

MTZ degradation follows pseudo first-order reaction kinetic equation (the unit, s^−1^) in the UV/H_2_O_2_ system, as follows:

(17) ktot [MTZ]=kUV[MTZ]+kMTZ,OH • [OH •]SS [MTZ]

Under steady-state conditions, the production rate (r0,OH •) of ^•^OH is equal to the consumption rate. Therefore, the steady-state concentration of ^•^OH (i.e., [OH •]SS) can be calculated by the following formula:

(18) 0=d[OH •]dt=r0,OH •−(kMTZ,OH • [MTZ][OH •]SS+k1[H2O2][OH •]SS+k2[HO2−][OH •]SS+kH1[OH •]SS[H2PO4−]+kH2[OH •]SS[HPO42−]+kHi[OH •]SS[Si])

In the UV/H_2_O_2_ system, r0,OH • can be calculated by the following formula [59]:

(19) r0,OH •=2φOH •EH=2φOH •I0 fH2O2 (1−10−A)

(20) $$f_{H_{2}O_{2}}=\frac{\;l\;\varepsilon_{H_{2}O_{2}}\left[H_{2}O_{2}\right]}{A}$$

where φOH • is the quantum yield (0.5 mol Einstein^−1^) of H_2_O_2_ at 254 nm [59]. The mean values of r0_OH • and $k_{\mathrm{tot}}$ were 2.34 × 10^−8^ M s^−1^ and 2.45 × 10^−3^ s^−1^ (pH 3.00).

Through the above formula, kMTZ,OH • and steady state concentration [OH •]SS was calculated by the following formula:

(21) kMTZ,OH •=(ktot−kUV)(k1[H2O2]+k2[HO2−]+kH1[H2PO4−]+kH2[HPO42−]+kHi[Si])2φHI0fH (1−10−l∑εiCi)−(ktot−kUV) [MTZ]

(22) [OH •]SS=2φHI0fH (1−10−l∑εiCi)k1[H2O2]+k2[HO2−]+kMTZ,OH •[MTZ]+kH1[H2PO4−]+kH2[HPO42−]+kHi[Si]

According to the steady state kinetic model, the kMTZ,OH • can be calculated as 2.43 (±0.25) × 10^9^ M^−1^ s^−1^, which is similar to the value determined by the competitive kinetic method in this study (2.79 (±0.32) × 10^9^ M^−1^ s^−1^). This verifies the reliability of this steady state kinetic model. Under the initial conditions of pH 3.00, the mean values of [OH •]SS were 2.36 × 10^−13^ M. Kwon et al. [47] reported that [OH •]SS was 2.70 × 10^−13^ M in their UV/H_2_O_2_ system ([H_2_O_2_]_0_ = 0.5 mM, *I*_0_ = 0.5 mW cm^−2^, *l* = 0.79 cm, [IBU]_0_ = 10 µM, pH = 7.00), which was consistent with our measured [OH •]SS value.

The contribution of direct photolysis and ^•^OH to MTZ degradation in the UV/H_2_O_2_ system can be calculated by the above $k_{{\mathrm{HO}}^{•},\mathrm{MTZ}}$, [OH •]SS and Formulas (21) and (22).

(23) rtot(rtotrtot)=rUV (rUVrtot)+rOH • (rOH •rtot)

At pH 3.00, *r*_UV_ and rOH • were 2.43 × 10^−9^ M s^−1^ and 2.21 × 10^−8^ M s^−1^ in the UV/H_2_O_2_ system, respectively.

rtot(100%)=rUV (9.9%)+rOH • (90.1%)

The contribution of direct photolysis and ^•^OH to the MTZ degradation is shown in the above. When the pH value is 3.00, the contribution of ^•^OH to MTZ degradation is 90.1%, while the contribution of direct photolysis is only 9.9%. The kinetic model results show that ^•^OH plays a major role in the MTZ degradation and ^•^OH is the main active material in the UV/H_2_O_2_ system. This result also verifies the conclusion that ^•^OH plays a major role in the MTZ degradation in UV/H_2_O_2_ system proposed in the kinetic analysis section.

### 3.6. Influence of H_2_O_2_ Dosage and Complex Matrix

#### 3.6.1. Effect of the Initial H_2_O_2_ Dosage

In the UV/H_2_O_2_ system, the pseudo first-order reaction kinetic model can be used to simulate and study the effects of other factors on MTZ degradation [41]. The contribution rate of direct photolysis to the degradation of MTZ ($k_{\mathrm{cal},\mathrm{UV}}$) and the contribution rate of ^•^OH to the degradation of MTZ (kcal,OH •) under different conditions are calculated, respectively, by the following formula:

(24) $$k_{\mathrm{cal},\mathrm{UV}\;}\;=\varphi_{\mathrm{MTZ}}I_{0}\frac{\;{l\;\varepsilon }_{\mathrm{MTZ}}}{A}(1-10^{-A})$$

(25) kcal,OH •=kMTZ,OH • [OH •]SS

The total contribution rate ($k_{\mathrm{cal},\mathrm{obs}}$) is the sum of direct photolysis and ^•^OH to MTZ degradation, as follows:

(26) kcal,obs=kcal,UV+kcal,OH •

**Figure 6:**
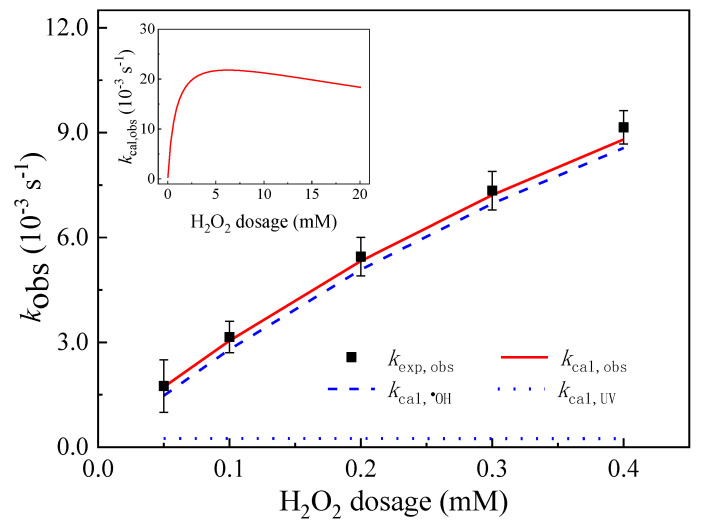
Impacts of H_2_O_2_ dosages on the pseudo first-order constants of MTZ (*k*_obs_). ([MTZ]_0_ = 10 μM, pH = 3.00, and *I*_0_ = 7.50 × 10^−6^ Einstein L^−1^ s^−1^, *l* = 0.935 cm.)

H_2_O_2_ dosage has an important effect on the degradation of MTZ in the UV/H_2_O_2_ system. As shown in Figure 6, with the H_2_O_2_ concentration gradually increases from 50 to 400 μM, the pseudo first-order reaction kinetic constant ($k_{\exp,\mathrm{obs}}$) of MTZ degradation actually measured increases from 1.75 × 10^−3^ s^−1^ to 9.15 × 10^−3^ s^−1^. The predicted value of kinetic model $k_{\mathrm{cal},\mathrm{obs}}$ and actual measured value $k_{\exp,\mathrm{obs}}$ coincided well.

The contribution rate of direct photolysis and ^•^OH to the MTZ degradation calculated by the kinetic model shows that the concentration of H_2_O_2_ gradually increases from 50 to 400 μM and the contribution rate of ^•^OH to the MTZ degradation is always greater than 90%, while the contribution rate of direct photolysis is less than 10%. ^•^OH is always the main active material in the UV/H_2_O_2_ system and plays a major role in the MTZ degradation. The kinetic model predicts that with an initial H_2_O_2_ concentration of 90 μM in the UV/H_2_O_2_ system, the contribution rates of ^•^OH and direct photolysis to MTZ degradation are 92.68% and 7.32%, which are basically consistent with the actual measured contribution rates of ^•^OH and direct photolysis (90.1% and 9.9%). Guo et al. [71] found that with the gradual increase in H_2_O_2_ concentration from 0 to 5 mM, the experimentally determined apparent rate constant ($k_{\mathrm{app}}$) of ciprofloxacin gradually increased from 0.39 × 10^−3^ s^−1^ to 3.72 × 10^−3^ s^−1^, which is consistent with the results obtained in our study. The kinetic model simulation results also show that with the further increase in H_2_O_2_ concentration, it would have a negative impact on the degradation of MTZ. As shown in the internal diagram of Figure 6, when the H_2_O_2_ concentration exceeds 5 mM, $k_{\mathrm{cal},\mathrm{obs}}$ gradually showed a downward trend. The main reason for this is that ^•^OH can be cleared by excess H_2_O_2_, and the second-order reaction rate constant between ^•^OH and H_2_O_2_ is 2.7 × 10^7^ M^−1^ s^−1^ [60].

#### 3.6.2. Effect of Organic Matter

**Figure 7:**
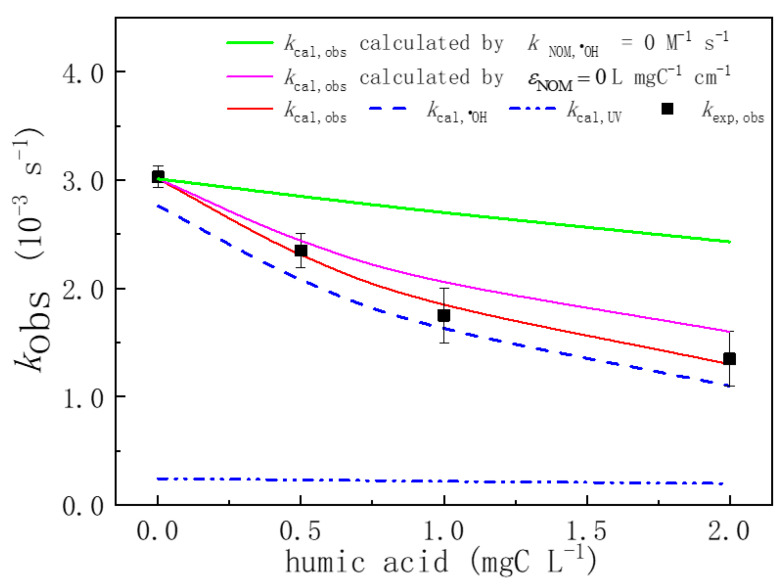
Impacts of humic acid on the pseudo first-order constants of MTZ (*k*_obs_). ([MTZ]_0_ = 10 μM, [H_2_O_2_]_0_ = 100 μM, and *I*_0_ = 7.50 × 10^−6^ Einstein L^−1^ s^−1^, *l* = 0.935 cm).

As humic acid is the main component of organic matter, the effect of organic matter on the degradation of MTZ in the UV/H_2_O_2_ system can be studied by adding humic acid of different concentrations. Figure 7 shows that the humic acid concentration increases from 0 ^1^ to 2.0 mgC L^−1^, $k_{\exp,\mathrm{obs}}$ gradually decreased from 3.01 × 10^−3^ s^−1^ to 2.43 × 10^−3^ s^−1^ at pH 3.00 condition. The $k_{\mathrm{cal},\mathrm{obs}}$ value of MTZ degradation can be obtained by model calculation in the UV/H_2_O_2_ system, which has added humic acid, and actual measured value $k_{\exp,\mathrm{obs}}$ is slightly larger than $k_{\mathrm{cal},\mathrm{obs}}$ calculated by the model, indicating that humic acid not only plays the role of quenching free radicals and competing for ultraviolet light in the system, but also contributes to the MTZ degradation by some secondary free radicals, which are generated by the reaction of humic acid and ^•^OH [46].

In this study, the inhibition rate of competing UV light and quenching free radicals on the MTZ degradation by humic acid was predicted by the kinetic model. After the addition of humic acid, the overall absorbance of the solution in the UV/H_2_O_2_ system is *A* (*A* = *l*(*ε*_MTZ_ [MTZ] +$\;\varepsilon_{H_{2}O_{2}}$ [H_2_O_2_] + *ε*_NOM_ [humic acid]), and *ε*_NOM_ is determined to be 0.10 L mgC^−1^ cm^−1^. If the competition of humic acid for ultraviolet light is ignored (i.e., assuming that the *ε*_NOM_ of humic acid is zero), $k_{\mathrm{cal},\mathrm{obs}}$ (purple) only obtained a slight increase (Figure 7). If the role of humic acid as a quenching radical is ignored (i.e., assuming no reaction between humic acid and ^•^OH), $k_{\mathrm{cal},\mathrm{obs}}$ is greatly improved compared with the actual measured value. These comparative studies show that the effect of humic acid as a quenching radical is greater than that of humic acid as competition for ultraviolet light on the inhibition of MTZ degradation. At the same time, this study compared the contribution rate of direct photolysis and ^•^OH to MTZ degradation in the UV/H_2_O_2_ system with humic acid. The results show that the contribution rate of ^•^OH was still significantly higher than that of direct photolysis, and ^•^OH still played a major role in MTZ degradation.

#### 3.6.3. Effect of Inorganic Anions

In the natural water environment, Cl^−^, ${\mathrm{SO}}_{4}^{2-}$, ${\mathrm{NO}}_{3}^{-}$ and ${\mathrm{HCO}}_{3}^{-}$ is the main inorganic anion. Therefore, the effect of inorganic anions on MTZ degradation was studied by adding different concentrations (0~5 mM) of Cl^−^, ${\mathrm{SO}}_{4}^{2-}$, ${\mathrm{NO}}_{3}^{-}$ and ${\mathrm{HCO}}_{3}^{-}$ in the UV/H_2_O_2_ system. At pH 3.00 condition, ${\mathrm{CO}}_{3}^{2-}{/\mathrm{HCO}}_{3}^{-}$ do not exist in the water body, so the impact of ${\mathrm{CO}}_{3}^{2-}{/\mathrm{HCO}}_{3}^{-}$ was not considered in this study. According to the actual measurement, the molar absorptivity of ${\mathrm{SO}}_{4}^{2-}$, ${\mathrm{NO}}_{3}^{-}$ and Cl^−^ at 254 nm is 0.31, 3.53 and 0.045 M^−1^cm^−1^, respectively. The study of Xiao et al. [41] also confirmed that the inorganic anions such as ${\mathrm{SO}}_{4}^{2-}$, ${\mathrm{NO}}_{3}^{-}$ and Cl^−^ have no effect on the absorbance value of the reaction solution at 254 nm. Since inorganic anions have no absorbance to UV in UV254 and have no influence on MTZ direct photolysis, direct photolysis rate after adding inorganic anions is approximately equal to the direct photolysis rate when no ions are added. Meanwhile, the production rate of ^•^OH can be regarded as approximately equal to that in deionized water. Therefore, the addition of inorganic anions mainly affects the degradation rate through the action of ^•^OH and a series of subsequent reactions.

**Figure 8:**
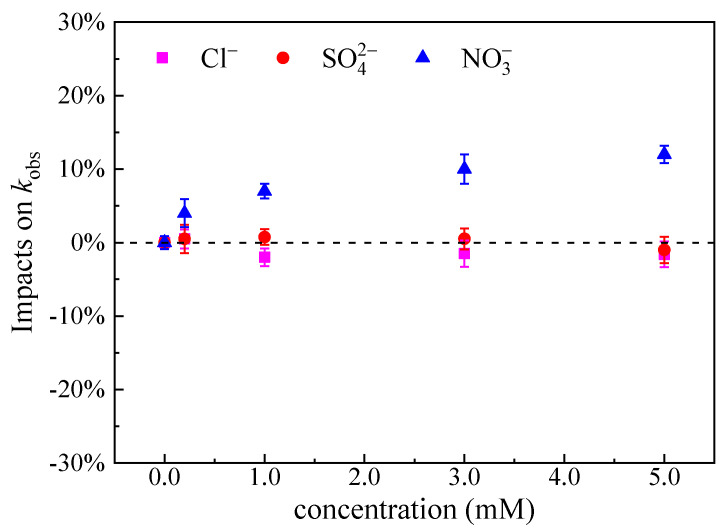
Impacts of inorganic anions on the pseudo first-order constants of MTZ (*k*_obs_). ([MTZ]_0_ = 10 μM, [H_2_O_2_]_0_ = 100 μM, and *I*_0_ = 7.50 × 10^−6^ Einstein L^−1^ s^−1^, *l* = 0.935 cm).

As shown in Figure 8, ${\mathrm{NO}}_{3}^{-}$ slightly promoted the MTZ degradation in the system, which is consistent with what Xiao et al. [41] reported. Xiao et al. [41] found that the degradation of CHCl_2_I was promoted by adding ${\mathrm{NO}}_{3}^{-}$ to the UV/H_2_O_2_ system. Under neutral conditions, the redox potential of ${\mathrm{NO}}_{3}^{•}$/${\mathrm{NO}}_{3}^{-}$ (2.3~2.6 V) is closed to that of ^•^OH/H_2_O (2.39 V), which leads to a slow reaction between ${\mathrm{NO}}_{3}^{-}$ and ^•^OH. Therefore, ${\mathrm{NO}}_{3}^{-}$ has limited scavenging effect on free radicals [41]. However, ${\mathrm{NO}}_{3}^{-}$ can absorb ultraviolet light and produce ^•^OH in a low quantum yield in the process of complex photolysis (Table 2) [69].

However, ${\mathrm{SO}}_{4}^{2-}$ and Cl^−^ had little effect on the degradation of MTZ in the UV/H_2_O_2_ system. This result is consistent with the previous findings of Xiao et al. [41] and Guo et al. [71]. Xiao et al. [41] found that the CHCl_2_I degradation rate did not change in the UV/H_2_O_2_ system by adding 1~5 mM ${\mathrm{SO}}_{4}^{2-}$ and Cl^−^. Guo et al. [71] also reported that ${\mathrm{SO}}_{4}^{2-}$ had no effect on the ciprofloxacin degradation. The redox potentials of ${\mathrm{SO}}_{4}^{•-}$/${\mathrm{SO}}_{4}^{2-}$ (2.43 V) and Cl^•^/Cl^−^ (2.41 V) are very similar to ^•^OH/H_2_O (2.39 V) under neutral conditions [72]. The results of this study show that the scavenging effect of ${\mathrm{SO}}_{4}^{2-}$ on free radicals is negligible and the scavenging effect of Cl^−^ on free radicals is limited. It reports that ^•^OH can react with Cl^−^ rapidly to form, for example, Cl^•^, ${\mathrm{ClOH}}^{•-}$, ${\mathrm{Cl}}_{2}^{•-}$ and other secondary chlorine radicals. ${\mathrm{ClOH}}^{•-}$ and ${\mathrm{Cl}}_{2}^{•-}$ can also generate ^•^OH and Cl^−^ through complex chain reaction under the pH condition greater than 7.2 [73]. Therefore, the reaction between the secondary free radicals generated by adding Cl^−^ to the UV/H_2_O_2_ system and MTZ cannot be ignored. For example, Cl^•^ is a strong selective oxidant with high reactivity to compounds containing aromatic groups and electron-rich groups [74]. ${\mathrm{Cl}}_{2}^{•-}$ and Cl^•^ have strong oxidation ability, and the redox potential is 2.0 and 2.47 V, respectively [75]. Studies have shown that in some cases, the reaction activity of ${\mathrm{Cl}}^{•}$ with organic substances is higher than that of ^•^OH. For example, ${\mathrm{Cl}}^{•}$ reacts faster with three substituted aromatic hydrocarbons, toluene, benzoic acid and chlorobenzene, compared to ^•^OH [76]. ${\mathrm{Cl}}_{2}^{•-}$ oxidizing is less than ${\mathrm{Cl}}^{•}$, but it can also react with organics selectively [77]. Therefore, the effect of Cl^−^ quenching free radicals is offset by the ^•^OH released by some intermediates (such as ClOH^•−^) and the reaction of these secondary free radicals with MTZ [78].

Natural water bodies contain a variety of complex matrices. The influence of the complex matrix in natural water on the degradation of organic pollutants could not be ignored. According to the previous reported works, organic matter and inorganic anions are the two substances with a relatively large impact on the removal of organic pollutants [46,69]. Similar results were found on MTZ degradation in the UV/H_2_O_2_ system. The results could provide theoretical guidance for the selection of removal technologies of organic pollutants and the specific application of advanced oxidation technologies based on ^•^OH in natural water bodies, such as rivers, lakes, and groundwater.

## 4. Conclusions

In this study, we systematically compared the MTZ degradation behavior and mechanism in the alone UV and UV/H_2_O_2_ systems. Specifically, we determined the effective light intensity and effective light path of the photochemical reaction system by using two chemical photometric methods of potassium ferric oxalate and hydrogen peroxide, and we found that the results are consistent in the two methods. MTZ degradation conformed to the pseudo first-order reaction kinetic equation in the UV and UV/H_2_O_2_ systems. Compared with the alone UV system, MTZ had a faster degradation rate in the UV/H_2_O_2_ system. Molar absorption coefficient and quantum yield have significant effects on the direct photolysis of compounds. Our experimental results show that the relatively low molar absorption coefficient and quantum yield restricted the MTZ direct photolysis. Then, we calculated kMTZ,OH • and [OH •]SS by establishing a kinetic model based on the steady-state hypothesis in our UV/H_2_O_2_ system. These measured values show good agreement with previously reported ones and competitive kinetic measurement results. Kinetic model analysis and the radical identification experiment showed that ^•^OH plays a major role in the MTZ degradation, and ^•^OH is the primary active material in the UV/H_2_O_2_ system. Finally, we predicted the effect of H_2_O_2_ dosage and the complex matrix through the above steady-state kinetic model. The predicted results show a good agreement with the experimental results. H_2_O_2_ dosage and the complex matrix have influence on MTZ degradation, which cannot be ignored. These results provided guidance for us to apply advanced oxidation technology based on ^•^OH in natural water bodies, such as rivers, lakes, and groundwater. In addition, the developed method combined with experimental and steady-state kinetic model approaches could be applicable to measure and predict the degradation kinetics for other antibiotics to prioritize persistent contaminants at the screening level.

## Figures and Tables

**Table 1:** UPLC method of MTZ and *p*CBA.

Compound	Acetonitrile	Phosphate Buffer (20 mM, pH 3)	λ	Flow Rate	Injection Volume	Column Temperature
			nm	mL min^−1^	µL	°C
MTZ	15%	85%	320	0.3	5	35
*p*CBA	30%	70%	238			

**Table 2:** Summary of the reactions and *k* in the UV/H_2_O_2_ system.

#	Reaction	*k* (M^−1^ s^−1^)	Reference
1	H2O2+hv→2 OH •	r0,OH •=2φHEH, s^−1^	[59]
2	H2O2+OH •→HO2•+H2O	*k*_1_ = 2.7 × 10^7^	[60]
3	OH •+HO2−→HO2•+OH−	*k*_2_ = 7.5 × 10^9^	[59]
4	$H_{2}O_{2}\;↔{\;H}^{+}{+\mathrm{HO}}_{2}^{-}$	*k*_3_ = 2.51 × 10^−12^	[59]
	In the presence of Phosphates		
5	H_3_PO_4_ ⇌ H^+^ + $H_{2}{\mathrm{PO}}_{4}^{-}$	p*K*_a1_ = 2.1 unitless	[61]
6	$H_{2}{\mathrm{PO}}_{4}^{-}$ ⇌ H^+^ + ${\mathrm{HPO}}_{4}^{2-}$	p*K*_a2_ = 7.2 unitless	[61]
7	${\mathrm{HPO}}_{4}^{2-}$ ⇌ H^+^ + ${\mathrm{PO}}_{4}^{3-}$	p*K*_a3_ = 12.3 unitless	[61]
8	O •H+H2PO4−→ HPO4•−+H2O	*k*_H1_ = 2.0 × 10^4^	[60]
9	O •H+HPO42−→ HPO4•−+OH−	*k*_H2_ = 1.5 × 10^5^	[60]
10	OH •+PO43− → PO42−•+OH−	*k*_H3_ < 1.5 × 10^7^	[60]
11	OH •+H3PO4 → H2PO4•+H2O	*k*_H4_ = 2.7 × 10^6^	[60]
	In the presence of NOM		[62]
12	O •H+NOM→products	*k*_14_ = 1.4 × 10^4^ L mgC^−1^ s^−1^	[62]
	In the presence of Cl^−^		
13	O •H+ Cl−→ ClOH•−	*k*_16_ = 4.3 × 10^9^	[63]
14	${\mathrm{Cl}}^{•}{+\mathrm{OH}}^{-}\to {\;\mathrm{ClOH}}^{•-}$	*k*_17_ = 1.8 × 10^10^	[64]
15	${\mathrm{Cl}}^{•}{+H}_{2}O\to {\;\mathrm{ClOH}}^{•-}{+H}^{+}$	*k*_18_ = 2.5 × 10^5^	[63]
16	ClOH•−→ Cl−+O •H	*k*_19_ = 6.0 × 10^9^	[63]
17	${\mathrm{ClOH}}^{•-}{+H}^{+}\to {\;\mathrm{Cl}}^{•}{+H}_{2}O$	*k*_20_ = 2.1 × 10^10^	[64]
18	${\mathrm{Cl}}^{•}{+\mathrm{Cl}}^{-}\to {\;\mathrm{Cl}}_{2}^{•-}$	*k*_21_ = 8.5 × 10^9^	[65]
19	${\mathrm{Cl}}_{2}^{•-}{+H}_{2}O\to {\;\mathrm{ClOH}}^{•-}{+H}^{+}{+\mathrm{Cl}}^{-}$	*k*_22_ = 1.3 × 10^3^	[66,67]
20	${\mathrm{Cl}}_{2}^{•-}{+\mathrm{OH}}^{-}\to {\;\mathrm{ClOH}}^{•-}{+\mathrm{Cl}}^{-}$	*k*_23_ = 4.5 × 10^7^	[68]
21	${\mathrm{Cl}}_{2}^{•-}{+\mathrm{Cl}}_{2}^{•-}\to {\;\mathrm{Cl}}_{2}{+2\mathrm{Cl}}^{-}$	*k*_24_ = 9.0 × 10^8^	[65]
	In the presence of Carbonates		
22	O •H+ HCO3−→H2O+CO3•−	*k*_27_ = 8.5 × 10^6^	[60]
23	O •H+ CO32−→OH−+CO3•−	*k*_28_ = 3.9 × 10^8^	[60]
	In the presence of ${\mathrm{NO}}_{3}^{-}$		
24	O •H+NO3−→OH−+NO3•	*k*_30_ < 1.0 × 10^5^	[69]
25	NO3−+H+→ hv O •H+NO2•	ΦO •H = 0.24 mol Einstein^−1^	[69]
26	${\mathrm{NO}}_{3}^{-}+\mathrm{hv}\to {\mathrm{NO}}_{2}^{-}+\frac{1}{2}O_{2}$	$\Phi_{{\mathrm{NO}}_{2}^{-}}$	[70]
27	${\mathrm{NO}}_{3}^{-}+\mathrm{hv}\to {\mathrm{NO}}_{2}^{•}+O^{•-}$	*/*	[70]
28	${2\mathrm{NO}}_{2}^{•}{+H}_{2}O\to {\mathrm{NO}}_{2}^{-}+\;{\mathrm{NO}}_{3}^{-}+{2H}^{+}$	*/*	[69]
29	12O2+H2O →2OH •	*/*	[69]
30	O•−+H2O →OH •+OH−	*/*	[69]
	Degradation of MTZ		
31	MTZ+hv→products	*r*_UV_, M s^−1^	*
32	O •H+MTZ→products	$k_{{\mathrm{MTZ},\mathrm{HO}}^{•}\;}$	*
33	Secondary radical + MTZ →?	N.A.	N.A.

Note: ${\;\Phi }_{{\mathrm{NO}}_{2}^{-}}$ = 0.015~0.028 mol Einstein^−1^, * measure in this study.

## Data Availability

The authors confirm that the data supporting the findings of this study are available within the article.
